# Tumour Relapse Prediction Using Multiparametric MR Data Recorded during Follow-Up of GBM Patients

**DOI:** 10.1155/2015/842923

**Published:** 2015-08-27

**Authors:** Adrian Ion-Margineanu, Sofie Van Cauter, Diana M. Sima, Frederik Maes, Stefaan W. Van Gool, Stefan Sunaert, Uwe Himmelreich, Sabine Van Huffel

**Affiliations:** ^1^Department of Electrical Engineering (ESAT), STADIUS Center for Dynamical Systems, Signal Processing and Data Analytics, KU Leuven, Kasteelpark Arenberg 10, P.O. Box 2446, 3001 Leuven, Belgium; ^2^iMinds Medical IT, 3000 Leuven, Belgium; ^3^Department of Radiology, University Hospitals of Leuven, Herestraat 49, 3000 Leuven, Belgium; ^4^Department of Electrical Engineering (ESAT), PSI Center for Processing Speech and Images, KU Leuven, Kasteelpark Arenberg 10, 3001 Leuven, Belgium; ^5^Department of Pedriatic Neuro-Oncology, University Hospitals Leuven, Herestraat 49, 3000 Leuven, Belgium; ^6^Biomedical MRI/MoSAIC, Department of Imaging and Pathology, KU Leuven, 3000 Leuven, Belgium

## Abstract

*Purpose*. We have focused on finding a classifier that best discriminates between tumour progression and regression based on multiparametric MR data retrieved from follow-up GBM patients.* Materials and Methods*. Multiparametric MR data consisting of conventional and advanced MRI (perfusion, diffusion, and spectroscopy) were acquired from 29 GBM patients treated with adjuvant therapy after surgery over a period of several months. A 27-feature vector was built for each time point, although not all features could be obtained at all time points due to missing data or quality issues. We tested classifiers using LOPO method on complete and imputed data. We measure the performance by computing BER for each time point and wBER for all time points.* Results*. If we train random forests, LogitBoost, or RobustBoost on data with complete features, we can differentiate between tumour progression and regression with 100% accuracy, one time point (i.e., about 1 month) earlier than the date when doctors had put a label (progressive or responsive) according to established radiological criteria. We obtain the same result when training the same classifiers solely on complete perfusion data.* Conclusions*. Our findings suggest that ensemble classifiers (i.e., random forests and boost classifiers) show promising results in predicting tumour progression earlier than established radiological criteria and should be further investigated.

## 1. Introduction

GBM is the most common and malignant intracranial tumor [1], representing as much as 30% of primary brain tumors with increasing incidence in some geographic regions [2]. The patients have a median survival of only 10 to 14 months after diagnosis with only 3 to 5% of patients surviving more than three years. Recurrence is universal, and, at the time of relapse, the median survival is only five to seven months despite therapy [3].

The current standard of care is surgical resection followed by radiotherapy and concomitant and adjuvant temozolomide (TMZ) chemotherapy [4].

Magnetic resonance imaging (MRI) is the most widely used medical imaging technique for identifying the location and size of brain tumours. However, conventional MRI has a limited specificity in determining the underlying type of brain tumour and tumour grade [5, 6]. More advanced MR techniques like diffusion-weighted MRI, perfusion-weighted MRI, and chemical shift imaging (CSI) are promising in the characterization of brain tumours as they give potentially more physiological information [7–9].

Diffusion-weighted imaging (DWI) and diffusion kurtosis imaging (DKI) visualize the tissue structure and are useful for assessing tumour cellularity, because they give information about the movement of the water inside different tissues including biological barriers. Typical parameters related to diffusion are apparent diffusion coefficient (ADC), mean diffusivity (MD), mean kurtosis (MK), and fractional anisotropy (FA). MD is a general parameter that accounts for the mean diffusivity in all directions, MK might be a specific parameter for tissue structure [10], and FA is a general index of anisotropy, with a value of zero corresponding to isotropic diffusion and a value of one corresponding to diffusion only in one direction.

Perfusion-weighted MRI (PWI) provides measurements that reflect changes in blood flow, volume, and angiogenesis. Hypervascularity due to glioma-induced neoangiogenesis may show up as high relative cerebral blood volume (rCBV) while necrosis of different tissues may show up as low rCBV [11].

MR spectroscopy provides information about metabolites present in normal and abnormal tissues [12]. This information can be represented as metabolite maps using CSI.

We have studied patients with GBM that had the tumour surgically removed and afterwards were treated according to two different protocols developed for evaluating dendritic cell immunotherapy: HGG-IMMUNO-2003 [13–16] and HGG-IMMUNO-2010 [13].

The focus of our paper is finding a map between the multiparametric MR data acquired during the follow-up of the patients and the relapse of the brain tumour after surgery, as described by the clinically accepted RANO criteria [17]. In order to do this, we test different families of classifiers on multiparametric MR data, starting from simple ones, for example, *k*-nearest neighbours (*k*-NN) and linear discriminant analysis (LDA), and moving to nonlinear classifiers, for example, random forests and neural networks, using a total of 27 features extracted from PWI, DKI, and CSI data.

## 2. Materials and Methods

### 2.1. Study Setup

There are 29 patients included in this study, out of which 16 patients were treated according to the HGG-IMMUNO-2003 protocol [13–16] and 13 patients according to the HGG-IMMUNO-2010 protocol [13].

Patients that were treated according to the HGG-IMMUNO-2003 protocol are patients with relapsed GBM that received immune therapy as the sole treatment strategy.

Patients that were treated according to the HGG-IMMUNO-2010 protocol are patients with primary GBM that had surgery. For the follow-up treatment after surgery the patients were split into two groups. The first group consisting of 6 patients who received radiochemotherapy and the immune therapy vaccine. The second group consisting of the remaining 7 patients who received just radiochemotherapy for the first six months after surgery, and after those six months all 7 patients received radiochemotherapy plus the immune therapy vaccine. We refer to the first group as “HGG-IMMUNO-2010 vaccine” and to the second group as “HGG-IMMUNO-2010 placebo.”

All 29 patients were offered monthly MRI follow-up, but after six months under immune therapy all patients switched to a three-monthly schedule.

The local ethics committee approved this study and informed consent was obtained from every patient before the first imaging time point.

Based on radiological evaluation of the follow-up MRI scans using the current guidelines for response assessment of high grade glioma [17], each patient was assigned to one of two clinical groups:

- patients with* progressive disease* during follow-up which exhibit an increase of ≥25% in the sum of the products of perpendicular diameter of enhancing lesions compared to the smallest tumour measurement obtained either at baseline or best response,
- patients with* complete response* with disappearance of all measurable and nonmeasurable disease sustained for at least 4 weeks.

Based on this assessment, each MRI time point for each patient was considered to be labeled or unlabeled as follows: labeled as “responsive” for all time points at and after the moment when the patient was considered as “complete response”; labeled as “progressive” for all time points at and after the moment when the patient was considered as “progressive disease”; or “unlabeled” for all time points preceding the decision moment.

### 2.2. MRI Acquisition and Processing

Magnetic resonance imaging was performed on a clinical 3 Tesla MR imaging system (Philips Achieva, Best, Netherlands), using a body coil for transmission and a 32-channel head coil for signal reception. The imaging protocol consisted of diffusion kurtosis imaging, dynamic susceptibility weighted contrast-MRI (DSC-MRI), and MR spectroscopy, combined with standard anatomical imaging (T1-weighted MRI after contrast administration, T2-weighted MRI, and FLAIR (fluid attenuated inversion recovery) MR images).

#### 2.2.1. Anatomical Magnetic Resonance Imaging

MR images were acquired as previously described [9, 18, 19]. In brief, an axial spin echo T2-weighted MR image (TR/TE: 3000/80 msec, slice/gap: 4/1 mm, field of view (FOV): 230 × 184 mm^2^, turbo factor (TF): 10, and acquisition matrix: 400 × 300), an axial fluid-attenuated inversion recovery (FLAIR) image (TR/TE/IR: 11000/120/2800 msec, slice/gap: 4/1 mm, and acquisition matrix: 240 × 134), and a T1-weighted 3D spoiled gradient echo scan (fast field echo-FFE, TR/TE: 9.7/4.6 msec, flip angle: 8°, turbo field echo factor: 180, acquisition voxel size: 0.98 × 0.98 × 1 mm^3^, 118 contiguous partitions, and inversion time: 900 msec) after contrast administration were acquired as high-resolution anatomical reference images.

Regions of interest (ROI) were manually drawn around the solid contrast-enhancing region if present, avoiding areas of necrosis (N) or cystic components such as the surgical cavity. A second ROI was manually drawn around the entire lesion (TO), that is, contrast enhancement (CE) and perilesional oedema (ED). The ROI containing the perilesional oedema was obtained by extracting the contrast-enhancing portion from the total lesion. Finally, a separate ROI was drawn around the contralateral normal appearing white matter (NAWM) to standardize the hemodynamic measurements of DSC-MRI.

**Figure 1:**
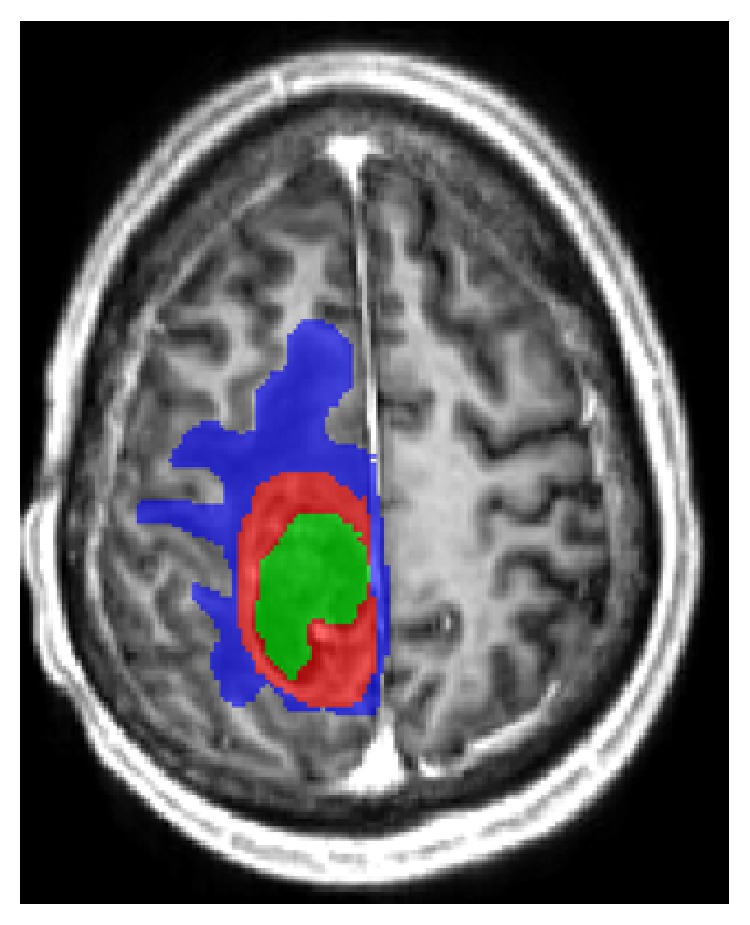
Delineations on T1 MR image postcontrast. Green—necrosis, red—CE, and blue—ED.

The manual delineations were drawn by a radiologist (SVC) with 5 years experience of MR imaging of brain tumours. An example of delineations on T1 post contrast image can be seen in Figure 1, where green is the necrosis, red is CE, and blue is ED.

#### 2.2.2. Magnetic Resonance Spectroscopy

A 2D-CSI short echo time protocol was used as validated in [20]. The volume of interest (VOI) is positioned on the slice of the transverse reconstruction of the T1-weighted 3D-FFE sequence with the largest section of contrast enhancement. The slice thickness of the VOI is 10 mm and the VOI is 80 × 80 × 10 mm^3^, with each voxel being 5 × 5 × 10 mm^3^ (16 × 16 voxels in total). If the contrast-enhancing lesion was smaller than 2 cm^3^ or the contrast enhancement is located in areas with large susceptibility differences, for example, the basal forebrain or the anterior temporal lobes, a single voxel (SV) technique was performed (TR/TE: 2000/35 msec, minimal volume: 1 cm^3^).

MR spectra were processed using the MATLAB 2010b environment (MathWorks, MA, USA) with SPID graphical user interface [21] as described in detail in [20].

Nine metabolites were quantified using the AQSES-MRSI quantification method [22]: N-acetyl aspartate (NAA), glutamine (Gln), glutamate (Glu), total creatine (Cre), phosphorylcholine (PCh), glycerophosphorylcholine (GPC), myo-inositol (Myo), and lipids (Lips) at 0.9 and 1.3 ppm, referred to as Lip1 and Lip2, respectively. Glu + Gln and PCh + GPC were reported as Glx and tCho (total choline), respectively. For each metabolite, AQSES-MRSI reported metabolite concentrations in institutional units and their error estimates as Cramer-Rao lower bounds (CRLBs) [23]. After quantification, good quality voxels were selected based on the CRLBs and spectral quality assessment as recommended by Kreis (FWHM of metabolites <0.07–0.1 ppm, no unexplained features in the residuals, no doubled peaks or evidence for movement artifacts, symmetric line shape, no outer volume ghosts or other artifacts present) [24]. CRLB lower than 20% for tCho, NAA, Glx, Cre, and Lips and CRLB lower than 50% for Myo were considered sufficient. From these representative voxels, the mean metabolite ratios as proposed by Kounelakis et al. were calculated [25] over the CE region: NAA/tCho, NAA/sum, tCho/sum, NAA/Cre, Lips/tCho, tCho/Cre, Myo/sum, Cre/sum, Lips/Cre and Glx/sum (10 parameters). The sum represents the sum of the concentrations of all quantified metabolites.

Sixty-six percent (66%) of all spectroscopic time points are not included in this study. There are two reasons for this: (1) quantification was not possible for all time points (MR spectroscopy data was not acquired for all patients due to patient movement) and (2) the rest of them did not pass the quality control recommended by Kreis [24].

#### 2.2.3. Dynamic Susceptibility Weighted Imaging (DSC-MRI)

Perfusion images were obtained using a standard DSC perfusion MR imaging protocol consisting of a gradient echo-EPI sequence, TR/TE: 1350/30 msec, section thickness/gap: 3/0 mm, dynamic scans: 60, FOV: 200 × 200 mm^2^, matrix: 112 × 109, number of slices: 23, and scan time: 1 minute 26 seconds. EPI data were acquired during the first pass following a rapid injection of a 0.1 mmol/kg body weight bolus of megluminegadoterat (Dotarem, Guerbet, Villepinte, France) via a mechanical pump at a rate of 4 mL/sec, followed by a 20 mL bolus of saline. Preload dosing was performed according to Hu et al. in order to correct for T1-weighted leakage (preload dose 0.1 mmol/kg megluminegadoterat, incubation time 10 min) [26].

DSC data were analyzed using DPTools (http://www.fmritools.org), as described in [18].

The mean values of the considered perfusion parameters were retrieved in the CE, ED, and NAWM regions. We report relative rCBV (rrCBV), relative rCBF (rrCBF), and relative DR (rDR) of tumoural tissue by using the corresponding parameter value in the contralateral NAWM as internal reference.

Although quantification was possible for all time points, after quality assessment done by visual inspection by SVC, 30% of them were not included in this study.

#### 2.2.4. Diffusion Kurtosis Imaging (DKI)

DKI data were acquired according to the previously described protocol in [18, 19] (SE-EPI-DWI sequence with TR/TE: 3200/90 msec, *δ*/Δ: 20/48.3 msec; FOV: 240 × 240 mm^2^, matrix: 96 × 96, number of slices: 44, 1 signal average acquired, section thickness/gap: 2.5/0 mm, and *b*-values: 700, 1000, and 2800 sec/mm^2^ in 25, 40, and 75 uniformly distributed directions, resp.) [27]. The DKI data were processed as described in [18]. Fractional anisotropy (FA), mean diffusivity (MD), and mean kurtosis (MK) were derived from the tensors [10, 28]. A nonlinear registration of the parameter maps to the anatomical MR imaging data was performed to minimize the local misalignment between the EPI distorted DKI data and the anatomical data on which the ROIs were manually positioned. MK, MD, and FA were determined in the CE and ED regions.

Although quantification was possible for all time points, after quality control according to [27], 44% of them were not included in this study.

#### 2.2.5. Summary of MRI Acquisition and Processing

In total, from 29 patients, we have 178 data points of follow-up MR imaging sessions, and each of these ones has 27 features:

- 3 volumes, contrast enhancement (CE), oedema (ED), and necrosis (N)
- 6 perfusion features, rrCBV, rrCBF, and rDR for CE and ED
- 6 diffusion features, MK, MD, and FA for CE and ED
- 10 spectroscopic features, from CE—NAA/tCho, NAA/sum, tCho/sum, NAA/Cre, Lips/tCho, tCho/Cre, Myo/sum, Cre/sum, Lips/Cre, and Glx/sum
- a parameter (0 or 1) for total resection of the tumour
- a parameter (0, 1, or 2) to describe the group of the patient, HGG-IMMUNO-2003, HGG-IMMUNO-2010 placebo, or HGG-IMMUNO-2010 vaccine.

Out of all 178 measurements, if we extract just the ones with complete features, it will result in a subset of 18 patients with 45 measurements. This implies that more than 75% of the measurements have at least one feature missing. Five features are always present: the three volumes, the parameter for tumour resection, and the parameter for different groups.

### 2.3. Classifiers

We have used several supervised and semisupervised classifiers, as presented in Table 1, with the goal of testing whether the unlabeled data could have been reliably labeled before the actual labeling was performed in the clinic according to the RANO criteria.

The list of classifiers in Table 1 is representative for the most important families of classification methods, starting from simple classical methods such as linear discriminant analysis (LDA) and *k*-nearest neighbour (*k*-NN) up to more complex nonlinear classifiers such as random forests and neural networks.

Each classifier is based on a mathematical model, which needs to be optimised on the basis of a training dataset. The training set consists here of labeled data, that is, data at and after a clinical decision has been made. The test set on which we compare classifiers consists of data that have no label, that is, time points before the decision of “progressive” or “responsive” has been made.

All classifiers are implemented in MATLAB R2013a (MathWorks, MA, USA). All classifiers except least squares support vector machines (LSSVMs) and the semisupervised ones are part of the Statistics Toolbox and Neural Networks Toolbox of MATLAB R2013a.

*k*-NN [29] is one of the basic classifers in machine learning. The class label of a new testing point is given by the most common class among its *k* neighbours. We used the default MATLAB R2013a (Statistics Toolbox) function “*k*nnclassify” to run a grid search for the best combination of number of neighbours (*k*) and type of distance. We varied *k* between 1 and 11 and the distance was either “euclidean,” “cityblock,” “cosine,” or “correlation.” We found the best results for the combination of 3 neighbours and the “correlation” distance.

Diagonal LDA (dLDA [30]) is a simple modification of linear discriminant analysis, which implies that we use the pseudoinverse of the covariance matrix instead of the actual inverse. We used the default MATLAB R2013a implementation “classify” from the Statistics Toolbox.

SVMs [31, 32] are among the most popular machine learning models because they are easy to understand: given a training set with points that belong to two classes, we try to find the best hyperplane to differentiate between the two types of points. We can try this in the original space or we can map the points to another space by using the kernel trick. We used the default MATLAB R2013a (Statistics Toolbox) implementations “svmtrain” and “svmclassify.” We used different types of kernel: linear, polynomial, radial basis function, and multilevel perceptron.

Classification tree [33] is an algorithm commonly used in machine learning. Like in a real tree there are leaves which represent class labels and branches. At each node of a tree a single feature is used to discriminate between different branches. We used the default MATLAB R2013a (Statistics Toolbox) implementation “classregtree.”

Neural networks [34–37] are built on interconnected layers of artificial “neurons” that try to map an input vector to its specific output. There are three types of layers: input, hidden, and output. The weights between different neurons are trained until a maximum number of iterations or a minimum error is reached. We used the default MATLAB R2013a (Neural Network Toolbox) implementation “net” with 10 hidden neurons. We tested four types of neural networks: pattern net, feed forward net, cascade forward net, and fit net.

Random forests [38, 39] are part of the ensemble methods for classification that use a collection of decision trees. Each decision tree learns a rule and then it can classify a new point. The new point is assigned to the class voted by the majority of the decision trees. We used the default MATLAB R2013a (Statistics Toolbox) implementation “TreeBagger” with 100 trees.

Boosting algorithms [40–43] start with a collection of weak classifiers (e.g., decision trees) and with each iteration they try to improve the overall classification by learning what was misclassified at the previous step. We used the default MATLAB R2013a (Statistics Toolbox) implementation “fitensemble” with 100 trees. We tested seven types of boosting algorithms: AdaBoost, LogitBoost, GentleBoost, RobustBoost, LPBoost, TotalBoost, and RUSBoost.

LSSVMs [44, 45] are a powerful machine learning technique. We downloaded LSSVMlab from [46] and followed the instructions from [47] to tune the parameters. We used different types of kernel: linear, polynomial, radial basis function, and also the Bayesian approach on LSSVM.

The semisupervised classifiers used in this paper are low density separation (LDS [48]), squared-loss mutual information regularization (SMIR [49]), and safe semisupervised support vector machine (S4VM [50, 51]). In the last years there has been a steady increase in the use and development of semisupervised classifiers, as they take into account information from unlabeled data also, not just from labeled data. This makes them powerful machine learning tools. The implementation for semisupervised classifiers was downloaded from [52–54].

Classifiers were tested first with all features described in Section 2.2.5 taken as input, but then also by selecting subsets of the available features as input, that is, only the features pertaining to a single modality (perfusion, diffusion, and spectroscopy). Additionally, classifiers were tested first on the smaller dataset containing 45 time points with a complete set of features and then on the larger dataset containing 178 time points where missing values have been imputed according to Section 2.4, presented below.

### 2.4. In-House Imputation Method

Some classifiers have built-in strategies of handling missing values, but other classifiers do not handle missing values (see Table 1). This is why we developed our own in-house imputation method, so the handling of missing values will be the same for all classifiers.

Our method is based on the volumes of contrast enhancement and oedema regions, in the sense that if the volume of a tumour region is zero, that missing tissue is considered healthy tissue. If we have values of any modality (perfusion, diffusion, and spectroscopy) that are missing from CE or ED, and the volume of CE or ED corresponding to that measurement is zero, and then we assume that those missing values belong to a normal type of tissue. For perfusion, because we normalize every parameter to the normal appearing white matter value, the missing values will be replaced by 1's. For diffusion and spectroscopy, the missing values will be replaced by the average of the features taken over the measurements which were labeled as responsive, because we consider that these measurements are recorded from a healthy tissue. If we have missing values without association to zero volume for CE or ED, they will be replaced by the average taken over all the labeled measurements.

### 2.5. Performance Indices

*Leave One Patient Out (LOPO)*. Classifiers are trained on labeled data from all patients except one who is the test patient. Each patient in turn is selected as test patient. All time points that belong to the test patient are classified independently. Results for each classifier are averaged per time point over all patients relative to the time point at which the clinical decision was made.

This way of testing is intuitive from a medical point of view and provides us with information about how good is the classification when we approach the decision time. In this way we can look at the temporal evolution of the classification for each patient.

We compute the balanced error rate (BER) at each time point before and after the decision, using the clinical decision assigned to each patient as expected label for all time points of this patient. BER is computed as

(1) $$\begin{array}{c} {\text{BER}}_{i}=\frac{{\text{ERR}}_{i}^{\text{resp}}+{\text{ERR}}_{i}^{\text{prog}}}{2}, \end{array}$$

where

(2) ERRiresp=Number  of  responsive  patientsllmisclassified  as  progressive×Total  number  of  responsive  patients−1,ERRiprog=Number  of  progressive  patientsllmisclassified  as  responsive×Total  number  of  progressive  patients−1.

For each classifier we have a grand total of 17 time points, due to the fact that there are patients with up to 6 time points after the decision time point and there are others with up to 11 time points before the decision. In order to compare the classifiers by using just one error number instead of 17, we compute a weighted average for each classifier's time response. This performance measurement is denoted by “weighted BER (wBER)” in the Results section.

We use two sets of weights:

(i) one for the temporal response—the classifier should perform better when we approach the labeling time point and after it:
  (3) $$\begin{array}{c} W_{i}^{t}=1,\;\text{if}\;i\geq \text{decision}\;\text{time}\;\text{point},\\ W_{i}^{t}=1-\frac{0.5}{11}\cdot i,\;\text{if}\;i<\text{decision}\;\text{time}\;\text{point} \end{array}$$
(ii) one for patient population—the time points with more patients get a higher weight (see Table 14 from the Appendix):
  (4) $$\begin{array}{c} W_{i}^{p}=\frac{\text{Number}\;\text{of}\;\text{patients}\;\text{at}\;\text{time}\;\text{point}\;i}{\text{Total}\;\text{number}\;\text{of}\;\text{patients}}. \end{array}$$
 The equation of wBER is
  (5) $$\begin{array}{c} \text{wBER}=\frac{\sum_{}^{}W_{i}^{p}\cdot W_{i}^{t}\cdot {\text{BER}}_{i}}{\sum_{}^{}W_{i}^{p}\cdot W_{i}^{t}}. \end{array}$$

## 3. Results and Discussion

### 3.1. Results

#### 3.1.1. LOPO When Using All Modalities

Table 7 from the Appendix shows how different classifiers perform on complete and on imputed features when using all MR modalities.

We selected the best 6 classifiers (marked by bold font in Table 7) and present their detailed BER results for each time point in Table 2.

Table 3 shows the detailed BER results for each time point for the best 6 classifiers (marked by bold font in Table 7) when using data with imputed features.

#### 3.1.2. LOPO When Using Each Modality

Table 4 shows how the best 6 supervised classifiers (marked by bold font in Table 7) perform on complete features when using each MR modality separately.

Tables 8, 9, and 10 from the Appendix list the performance of the best supervised classifiers (marked by bold font in Table 7) when using, respectively, perfusion, diffusion, or spectroscopy data separately, considering complete features only.

Table 5 shows how the best 6 classifiers (marked by bold font in Table 7) perform on imputed features when using each MR modality separately.

Tables 11, 12, and 13 from the Appendix list the performance of the best supervised classifiers (marked by bold font in Table 7) when using, respectively, perfusion, diffusion, or spectroscopy data separately, considering imputed features only.

#### 3.1.3. In-House Imputation Strategy versus Built-In Imputation Strategy

Table 6 shows how different classifiers perform with our in-house imputation of missing values (Section 2.4) versus the built-in imputation strategy of missing values for the classifiers marked in Table 1.

### 3.2. Discussion

A first conclusion that we can draw from a comparative analysis of the different classifiers is that we obtain the lowest error when training classifiers on data with complete features and not on data with imputed features, no matter the imputation method (our in-house method or the built-in method). In order to improve the performance of classifiers, improving the quality of the data would help.

The lowest error when using complete features is around 0.14 (SVM-mlp—0.136), while if we use imputed features the lowest error is 0.216 (dLDA). The best classifiers on complete features are ensemble classifiers (random forests and boosting algorithms), dLDA, and SVM, while the best classifiers on imputed features are dLDA, SVM-lin, and random forests.

If we compare the results of single MR modalities when training classifiers on data with complete features, we can say that the use of spectroscopy only leads to the worst results with a minimum error of 0.561. The single use of perfusion generates better results than using only diffusion data, especially when using ensemble methods (random forests, LogitBoost, and RobusBoost), with a minimum error of 0.148 compared to 0.255. When using imputed features, the minimum error almost doubles.

An interesting aspect when looking at detailed measurements on complete features (Table 2) is the fact that we have error equal to zero (perfect classification), one time point before the actual labeling according to the RANO criteria, when using random forests, LogitBoost, or RobustBoost. This means that we can predict the patient outcome (progressive, responsive) with 100% accuracy one time point (i.e., about 1 month in our study) earlier than the actual clinical decision was made. When looking at each MR modality separately (Tables 8, 9, and 10) we notice that the same result could have been obtained by using solely the perfusion data. This is a very important finding, mainly because perfusion is very fast to measure (2-3 minutes) and it has the lowest rate of missing data, which makes it reliable. Our study is not the only one that shows that perfusion parameters are very reliable when it comes to differentiating between tumour tissues and other tissues. Multiple studies (among others Barajas Jr. et al. [55] and Hu et al. [56]) prove that perfusion parameters are strongly correlated with tumour progression and overall survival. The main reason behind this strong correlation is the fact that tumours grow very fast, so they require large amounts of nutrients to develop, which is reflected in the angiogenesis of the tumour. This increase in angiogenesis is visualised and measured using perfusion imaging.

When comparing the two methods of imputing missing values, our in-house method (Section 2.4), and the classifier-dependent built-in strategies, the difference between them is not important with respect to the performance of the classifiers.

Using machine learning for classification of brain tumoral tissue is a field with an increasing amount of work.

In [57] Hu et al. use a support vector machine approach on multiparametric MRI (perfusion, diffusion, and anatomical MRI) to automatically differentiate between radiation necrosis voxels and progressive tumour voxels coming from patients with resected GBM. They optimize a one-class SVM based on the area under receiver operator curve from 6000 training voxels manually delineated from 8 patients and then tested on manually delineated voxels from 8 new patients. Their results show that perfusion and diffusion have a high discrimination rate between radiation necrosis and tumour progression.

In [55] Barajas Jr. et al. use perfusion MR imaging to investigate which parameters can be used to differentiate between recurrent GBM and radiation necrosis. Their study was based on 57 patients, they used Welch *t* test to compare measurements between groups, and they found that all perfusion parameters (relative CBV, peak height, and percentage of signal intensity recovery) are strongly correlated with tumour progression.

In [56] Hu et al. use perfusion metrics on contrast enhancement lesions (CBV mean, mode, maximum, width, and a new thresholding metric called fractional tumor burden (FTB)) to see how they correlate to overall survival (OS). Their study was based on 25 patients with recurrent GBM and found that all parameters are strongly correlated with OS.

In [58] Weybright et al. used chemical shift imaging (CSI) to differentiate voxels with tumour recurrence and radiation injury. Their study was based on 29 patients and they had high quality data for 28 of them (97%). They found that the Cho/NAA and Cho/Cr ratios may be the best numerical discriminators between tumour recurrence and radiation injury.

Although we cannot compare our results directly to the ones from the studies presented before due to different approaches on classifying different tissues, it is becoming more obvious that a learning algorithm based on multiparametric MR data will evolve in the near future and will help clinicians in differentiating between progressive tumoral tissue and other types (necrotic or normal).

## 4. Conclusions

In this paper we compare different supervised and semisupervised classifiers. We train them on multiparametric MR data with complete and imputed features. The data was acquired from 29 patients selected from follow-up studies of GBM. We investigate the leave-one-patient-out testing method and come to the conclusion that the same label according to the RANO criteria could have been put earlier with at least one month with 100% accuracy, if we train random forests, LogitBoost, or RobustBoost on data with complete features. More interesting is the fact that the same result is achieved by the same classifiers using only complete perfusion data.

For future work we plan on using the temporal evolution of the features when classifying different MR sessions and also allow updating the class labels in time. Moreover, we are going to try new methods of processing the raw MR data to improve the quality of it.

## Figures and Tables

**Table 1:** Supervised and semisupervised classifiers tested in this paper.

Supervised classifiers	Handles missing values
Random forests	✓
Classification tree	✓
Boost ensembles	✓
Neural networks	—
SVM	—
LSSVM	—
*k*-NN	—
dLDA	—

Semisupervised classifiers	

LDS	—
SMIR	—
S4VM	—

**Table 2:** Detailed BER results for each time point for the best 6 classifiers when using the leave-one-patient-out method on complete features for all MR modalities. The decision moment marked by bold font. Some time points do not have results because there were no complete measurements.

BER	Random forests	dLDA	SVM-lin	LogitBoost	RobustBoost	SVM-mlp
*L* + 5	—	—	—	—	—	—
*L* + 4	—	—	—	—	—	—
*L* + 3	0	0	0	0	0	0
*L* + 2	0	0	0	0	0	0
*L* + 1	0	0	0	0	0	0
**L**	**0 **	**0.1 **	**0.217**	**0**	**0**	**0.1**
*L* − 1	0	0.125	0	0	0	0.125
*L* − 2	0.25	0.25	0.5	0.25	0.25	0.25
*L* − 3	0.5	0.5	1	0.5	0.5	0.25
*L* − 4	1	1	1	1	1	0.5
*L* − 5	0.25	0.25	0.25	0.25	0.25	0.25
*L* − 6	0.5	0	0	0.5	0.5	0
*L* − 7	1	0	1	1	1	0
*L* − 8	—	—	—	—	—	—
*L* − 9	0	0	0	0	0	0
*L* − 10	—	—	—	—	—	—
*L* − 11	0	0	1	0	0	0

wBER	0.148	0.172	0.276	0.148	0.148	0.136

**Table 3:** Detailed BER results for each time point for the best 6 supervised classifiers when using the leave-one-patient-out method on imputed features for all MR modalities. The decision moment marked by bold font.

BER	Random forests	dLDA	SVM-lin	LogitBoost	RobustBoost	SVM-mlp
*L* + 5	0	0	0	0	0	0
*L* + 4	0	0	0	0	0	0
*L* + 3	0	0	0	0	0	0
*L* + 2	0.125	0.25	0.125	0.125	0.125	0
*L* + 1	0.171	0.071	0.071	0.171	0.171	0.071
**L **	** 0.105 **	**0.022 **	** 0.149 **	**0.188 **	**0.105 **	**0.359**
*L* − 1	0.214	0.065	0.130	0.3	0.192	0.192
*L* − 2	0.444	0.417	0.194	0.444	0.472	0.5
*L* − 3	0.418	0.382	0.282	0.418	0.418	0.482
*L* − 4	0.475	0.413	0.388	0.475	0.413	0.475
*L* − 5	0.688	0.438	0.563	0.688	0.688	0.688
*L* − 6	0.368	0.467	0.3	0.567	0.567	0.567
*L* − 7	0.375	0.375	0.75	0.5	0.75	0.625
*L* − 8	0.5	0.333	0.583	0.5	0.75	0.333
*L* − 9	0.333	0.333	0.833	0.333	0.833	0.5
*L* − 10	0.5	0.75	0.75	0.5	1	0.75
*L* − 11	0.5	0.5	1	0.5	0.5	0.5

wBER	0.294	0.216	0.242	0.335	0.325	0.352

**Table 4:** Weighted BER for the best 6 supervised classifiers when using the leave-one-patient-out method with complete features for each MR modality separately.

Weighted BER	Random forests	dLDA	SVM-lin	LogitBoost	RobustBoost	SVM-mlp
Perfusion	0.148	0.256	0.220	0.148	0.148	0.193
Diffusion	0.358	0.259	0.255	0.367	0.367	0.349
Spectroscopy	0.571	0.561	0.600	0.609	0.623	0.629

**Table 5:** Weighted BER for the best 6 supervised classifiers trained on imputed features from each MR modality separately.

Weighted BER	Random forests	dLDA	SVM-lin	LogitBoost	RobustBoost	SVM-mlp
Perfusion	0.294	0.311	0.275	0.289	0.265	0.282
Diffusion	0.277	0.327	0.322	0.277	0.277	0.380
Spectroscopy	0.412	0.401	0.423	0.423	0.408	0.415

**Table 6:** wBER comparison between our in-house method of imputing missing values and built-in imputation strategy of different supervised classifiers.

Weighted BER	Our method	Built-in method
Random forests	0.294	0.423
AdaBoost	0.324	0.333
LogitBoost	0.335	0.241
GentleBoost	0.308	0.245
RobustBoost	0.325	0.296
LPBoost	0.256	0.369
TotalBoost	0.289	0.323
RUSBoost	0.308	0.361
Decision tree	0.346	0.651

**Table 7:** Weighted BER for supervised and semisupervised classifiers trained on complete and imputed data. We marked the best 6 classifiers by bold font.

Weighted BER	Complete features	Imputed features	Average
dLDA	0.172	0.216	** 0.194 **
SVM-lin	0.276	0.242	** 0.259 **
SVM-poly	0.285	0.334	0.310
SVM-rbf	0.493	0.520	0.507
SVM-mlp	0.136	0.352	** 0.244**
Bayesian LSSVM	0.371	0.469	0.420
LSSVM-lin	0.452	0.280	0.366
LSSVM-poly	0.462	0.362	0.412
LSSVM-rbf	0.408	0.320	0.364
Random forests	0.148	0.294	** 0.221**
AdaBoost	0.505	0.324	0.415
LogitBoost	0.148	0.335	** 0.242 **
GentleBoost	0.296	0.308	0.302
RobustBoost	0.148	0.325	** 0.237**
LPBoost	0.505	0.256	0.381
TotalBoost	0.505	0.289	0.397
RUSBoost	0.281	0.308	0.295
Classification tree	0.268	0.346	0.307
3-NN (correlation)	0.357	0.428	0.392
Pattern net	0.449	0.288	0.366
Feed forward net	0.399	0.411	0.405
Cascade forward net	0.586	0.485	0.535
Fit net	0.535	0.350	0.443
LDS	0.442	0.534	0.488
SMIR	0.278	0.436	0.357
S4VM	0.456	0.473	0.465

**Table 8:** Detailed BER results for each time point for the best 6 supervised classifiers when using the leave-one-patient-out method on complete perfusion features. The decision moment marked by bold font. Some time points do not have results because there were no complete perfusion measurements.

BER on perfusion	Random forests	dLDA	SVM-lin	LogitBoost	RobustBoost	SVM-mlp
*L* + 5	—	—	—	—	—	—
*L* + 4	—	—	—	—	—	—
*L* + 3	0	0	0	0	0	0
*L* + 2	0	0	1	0	0	0
*L* + 1	0	0	1	0	0	0
**L **	0	0.217	0.05	**0 **	**0 **	**0.05 **
*L* − 1	0	0.187	0.187	0	0	0.187
*L* − 2	0.25	0.25	0.375	0.25	0.25	0.25
*L* − 3	0.5	0.5	0.5	0.5	0.5	0.5
*L* − 4	1	1	1	1	1	0.5
*L* − 5	0.25	0.25	0.25	0.5	0.5	0.5
*L* − 6	0.5	0.5	0.5	0.5	0.5	0.5
*L* − 7	1	1	1	1	1	1
*L* − 8	—	—	—	—	—	—
*L* − 9	0	0	0	0	0	0
*L* − 10	—	—	—	—	—	—
*L* − 11	0	0	0	0	0	0

**Table 9:** Detailed BER results for each time point for the best 6 supervised classifiers when using the leave-one-patient-out method on complete diffusion features. The decision moment marked by bold font. Some time points do not have results because there were no complete diffusion measurements.

BER on diffusion	Random forests	dLDA	SVM-lin	LogitBoost	RobustBoost	SVM-mlp
*L* + 5	—	—	—	—	—	—
*L* + 4	—	—	—	—	—	—
*L* + 3	0	0	0	0	0	1
*L* + 2	0	0.25	0	0	0	0.5
*L* + 1	0	0	0	0	0	0
**L **	0.217	0.1	0.1	0.217	0.217	0.267
*L* − 1	0.562	0.25	0.125	0.562	0.562	0.562
*L* − 2	0.5	0.25	0.5	0.5	0.5	0.375
*L* − 3	0.5	0.75	0.75	0.5	0.5	0.25
*L* − 4	0.5	1	0.5	0.5	0.5	0.5
*L* − 5	0.25	0.25	0.5	0.5	0.5	0
*L* − 6	0.5	0	0.5	0.5	0.5	0
*L* − 7	0	0	0	0	0	0
*L* − 8	—	—	—	—	—	—
*L* − 9	1	1	1	1	1	0
*L* − 10	—	—	—	—	—	—
*L* − 11	1	1	1	1	1	0

**Table 10:** Detailed BER results for each time point for the best 6 supervised classifiers when using the leave-one-patient-out method on complete spectroscopy features. The decision moment marked by bold font. Some time points do not have results because there were no complete spectroscopy measurements.

BER on spectroscopy	Random forests	dLDA	SVM-lin	LogitBoost	RobustBoost	SVM-mlp
*L* + 5	—	—	—	—	—	—
*L* + 4	—	—	—	—	—	—
*L* + 3	0	0	0	0	0	0
*L* + 2	1	0.75	0.75	1	1	1
*L* + 1	1	1	1	1	1	0
**L **	0.55	0.583	0.632	0.6	0.55	0.583
*L* − 1	0.562	0.562	0.813	0.5	0.562	0.687
*L* − 2	0.625	0.625	0.25	0.625	0.75	0.875
*L* − 3	0.25	0.5	0.25	0.5	0.5	0.25
*L* − 4	0.5	0.5	1	0.5	0.5	1
*L* − 5	0.5	0.5	0	1	1	1
*L* − 6	0.5	0	0.5	0.5	0.5	0.5
*L* − 7	0	0	1	0	0	0
*L* − 8	—	—	—	—	—	—
*L* − 9	1	1	1	1	1	0
*L* − 10	—	—	—	—	—	—
*L* − 11	1	1	1	1	1	0

**Table 11:** Detailed BER results for each time point for the best 6 supervised classifiers when using the leave-one-patient-out method on imputed perfusion features. The decision moment marked by bold font.

BER on perfusion	Random forests	dLDA	SVM-lin	LogitBoost	RobustBoost	SVM-mlp
*L* + 5	0	0	0	0	0	0
*L* + 4	0	0	0	0	0	0
*L* + 3	0	0.25	0	0	0	0.25
*L* + 2	0.125	0	0	0.125	0	0.125
*L* + 1	0.171	0.071	0.071	0.171	0.071	0
**L **	0.127	0.109	0.043	**0.127 **	**0.043 **	**0.109 **
*L* − 1	0.130	0.196	0.152	0.214	0.130	0.279
*L* − 2	0.444	0.528	0.472	0.389	0.444	0.417
*L* − 3	0.418	0.464	0.418	0.373	0.418	0.281
*L* − 4	0.475	0.475	0.475	0.412	0.475	0.512
*L* − 5	0.687	0.687	0.687	0.625	0.687	0.562
*L* − 6	0.567	0.567	0.567	0.567	0.567	0.567
*L* − 7	0.5	0.5	0.5	0.5	0.5	0.5
*L* − 8	0.5	0.5	0.5	0.5	0.5	0.5
*L* − 9	0.333	0.5	0.5	0.333	0.333	0.333
*L* − 10	0.5	0.5	0.5	0.5	0.5	0.25
*L* − 11	0.5	0.5	0.5	0.5	0.5	0

**Table 12:** Detailed BER results for each time point for the best 6 supervised classifiers when using the leave-one-patient-out method on imputed diffusion features. The decision moment marked by bold font.

BER on diffusion	Random forests	dLDA	SVM-lin	LogitBoost	RobustBoost	SVM-mlp
*L* + 5	0	0	0	0	0	0
*L* + 4	0	0	0	0	0	0
*L* + 3	0	0	0	0	0	0.25
*L* + 2	0	0.125	0	0	0	0
*L* + 1	0.1	0.243	0.243	0.1	0.1	0.314
**L**	**0.105**	**0.297**	**0.192**	**0.105 **	**0.105 **	**0.420 **
*L* − 1	0.254	0.257	0.257	0.254	0.254	0.424
*L* − 2	0.361	0.25	0.25	0.361	0.361	0.278
*L* − 3	0.282	0.473	0.473	0.282	0.282	0.436
*L* − 4	0.45	0.637	0.637	0.45	0.45	0.387
*L* − 5	0.562	0.5	0.562	0.562	0.562	0.437
*L* − 6	0.433	0.367	0.533	0.433	0.433	0.433
*L* − 7	0.5	0.5	0.5	0.5	0.5	0.75
*L* − 8	0.667	0.167	0.667	0.667	0.667	0.667
*L* − 9	0.667	0.667	0.667	0.667	0.667	0.5
*L* − 10	0.75	0.75	0.75	0.75	0.75	0.75
*L* − 11	1	0.5	1	1	1	1

**Table 13:** Detailed BER results for each time point for the best 6 supervised classifiers when using the leave-one-patient-out method on imputed spectroscopy features. The decision moment marked by bold font.

BER on spectroscopy	Random forests	dLDA	SVM-lin	LogitBoost	RobustBoost	SVM-mlp
*L* + 5	0	0	0	0	0	0
*L* + 4	0	0	0	0	0	0
*L* + 3	0	0	0	0	0	0.25
*L* + 2	0.25	0.25	0.125	0.25	0.25	0.25
*L* + 1	0	0	0	0	0	0
**L**	**0.562**	**0.504**	**0.609**	**0.587 **	**0.543 **	**0.569 **
*L* − 1	0.293	0.337	0.380	0.315	0.293	0.359
*L* − 2	0.389	0.389	0.389	0.389	0.389	0.389
*L* − 3	0.436	0.436	0.381	0.436	0.436	0.336
*L* − 4	0.55	0.55	0.612	0.55	0.55	0.55
*L* − 5	0.687	0.687	0.562	0.687	0.687	0.687
*L* − 6	0.433	0.433	0.533	0.6	0.433	0.433
*L* − 7	0.75	0.75	0.875	0.75	0.75	0.75
*L* − 8	0.667	0.667	0.667	0.667	0.667	0.667
*L* − 9	0.667	0.167	0.667	0.667	0.667	0.667
*L* − 10	0.75	0.75	0.25	0.75	0.75	0.75
*L* − 11	1	1	1	0.5	1	1

**Table 14:** Number of samples for each time point. The decision moment marked by bold font.

	Number of complete samples	Number of imputed samples
*L* + 5	0	2
*L* + 4	0	2
*L* + 3	1	3
*L* + 2	3	8
*L* + 1	1	12
**L**	** 13 **	** 29**
*L* − 1	9	29
*L* − 2	6	24
*L* − 3	3	16
*L* − 4	2	13
*L* − 5	2	12
*L* − 6	2	8
*L* − 7	1	6
*L* − 8	0	5
*L* − 9	1	4
*L* − 10	0	3
*L* − 11	1	2

## Appendix

In Tables 7–14 we use balanced error rate (BER) and weighted balanced error rate (wBER) to present the performance of the classifiers. BER and wBER are numbers between 0 and 1, 0 being perfect classification and 1 being total misclassification.
